# Computational Methods for Exploration and Analysis of Macromolecular Structure and Dynamics

**DOI:** 10.1371/journal.pcbi.1004585

**Published:** 2015-10-27

**Authors:** Amarda Shehu, Ruth Nussinov

**Affiliations:** 1 Department of Computer Science, George Mason University, Fairfax, Virginia, United States of America; 2 Department of Bioengineering, George Mason University, Fairfax, Virginia, United States of America; 3 School of Systems Biology, George Mason University, Fairfax, Virginia, United States of America; 4 Cancer and Inflammation Program, Leidos Biomedical Research, Inc., Frederick National Laboratory for Cancer Research National Cancer Institute, Frederick, Maryland, United States of America; 5 Sackler Institute of Molecular Medicine, Department of Human Genetics and Molecular Medicine, Sackler School of Medicine, Tel Aviv University, Tel Aviv, Israel

All processes that maintain and replicate a living cell involve fluctuating biological macromolecules. As computational biologists, our aim is to discern the behavior of macromolecules in a way that experimental biology is not able to achieve. No single technique—experimental or computational—can capture all the relevant scales of cellular functional behavior. In principle, computations are the tools that can integrate different kinds of experimental and computational characterizations at different resolutions to obtain a more complete description of the processes of life. Computer simulations can act as a bridge between the microscopic length and time scales, and the macroscopic world of the laboratory. They can start from a macroscopic experiment-based guess of interactions between molecules, and obtain “exact” predictions of bulk and detailed properties subject to limitations. They are able to test a theory by constructing and simulating the model, and comparing the results with experimental measurements; and they are able to provide models that experiments can test. Computations can provide leads by processing large sets of data, predicting molecular behaviors, and supplying the mechanistic underpinning that experiments alone may not be able to achieve.

Macromolecules play a vital role in countless biological processes, including DNA replication, transcription, genome reorganization in development and in disease, protein synthesis, protein folding, and active transport with molecular motors. Cell signaling, a multistep pathway on length scales from nanometers to micrometers, provides another inclusive example, incorporating all of the above over time and space. Signals are relayed from the extracellular space to the nucleus through dynamic shifts of molecular ensembles. Macromolecular fluctuations underlie signal amplification; they result in a large number of activated molecules across the cell, creating multiple reactions and producing a major cellular response. Changes in fluctuations through binding second messengers can regulate catalysis, and dynamic shifts in conformational ensembles can also take place through binding to membrane lipids. Key hub proteins that govern cell behaviors are often membrane-anchored. Helped by experimental data, computations can model the components of the systems and their transient interactions to provide a useful, integrated view of the flow of information, its regulation, and its deregulation.

Ultimately, we want to make direct quantitative comparisons with experimental data. We would like to reduce the amount of fitting and guesswork; but at the same time we may also be interested in phenomena of a generic nature, or in discriminating between good and bad theories. Doing this well is challenging. To understand the dynamic interplay across multiple scales, to link it to the atomic-scale physicochemical basis of the conformational behavior of single molecules and their interactions, and, ultimately, to relate it to cellular function, we need efficient and reliable methods to sample the macromolecular fluctuations and identify the biological and disease-related states and their transitions. Inspiration may come from a combination of biology and other fields that model dynamic systems.

Macromolecules move, and their movements are needed for a complete picture of life. Computational biology, with concepts imported from physics and chemistry, increasingly plays a major role, which has recently been recognized by the Nobel Committee [1]. The energy landscape underscores the inherent nature of biomolecules, which are dynamical objects that are always interconverting between structures with varying energies. It affirms that biomolecules must be described statistically, not statically. Macromolecules are not static objects; rather, they populate ensembles of conformations. The transitions between these states occur on length scales from tenths of an Ångström to nanometers, and time scales that can vary from nanoseconds to seconds. These are linked to functionally relevant phenomena such as allosteric signaling and enzyme catalysis.

Computational methods also include those for molecular modeling and refinement of three-dimensional structures, de novo design of proteins, prediction and modeling of protein-ligand interactions and development of docking protocols, and prediction of macromolecular interactions at varying spatial resolutions and timescales. They further encompass methods of ligand screening in drug development and protein-protein docking, methods for assessing sequence-structure-function relationships and prediction of macromolecular function, protocols for molecular visualization and annotation, and geometric and topological characterization of proteins and polynucleotides. This list is still far from complete.

To celebrate its tenth anniversary [2], *PLOS Computational Biology* presents a special collection of manuscripts focusing on methods exploring macromolecular structure and dynamics. This collection does not aim to cover all methods; it does, however, aim to provide a taste of currently available approaches and strategies toward these aims. Altogether, the collection covers a broad ground: from sampling, detection of rare events, and exposing hidden alternative backbone conformations in X-ray crystallography, to multi-scale visualization of molecular architecture using real-time ambient occlusion; from discrimination between obligatory and non-obligatory protein-protein interactions based on the dynamics of the complex to binding free energies of inhibitors, to predicting the effect of mutations on protein-protein interactions by exploiting interface profiles; from a virtual mixture approach to the study of multistate equilibrium, to identification of misfolded intermediates; from multiscale estimation of binding kinetics using a combination of Brownian dynamics, molecular dynamics, and mile-stoning, to mapping the protein fold universe. This collection underscores the breadth of computational methods in structural biology, and only some of them made it into this special *PLOS Computational Biology* collection.

Computational structural biology has made tremendous progress over the last two decades. Computational methods were developed for protein structure prediction, macromolecular function and protein design, as well as for drug discovery. It has also undertaken computational challenges related to experimental approaches in structural biology. Along with new experimental tools, higher resolution, and the rising efficiency of experimental approaches leading to huge amounts of accumulating data, computational biology is pushing the frontiers to meet its challenges. We expect that, in the future, the focus of our methods and tools may shift and more integrative tools will be developed, along with methodological adaptation to massively larger quantities of data.

Experimentally, the biological and chemical sciences are now attempting to push boundaries in drug discovery. We may expect that a translational direction will also prevail in computational structural biology. This, however, does not mean only direct drug discovery; for these efforts to be successful, the underlying mechanistic basis of diseases needs to be understood as well. In addition, we expect even stronger emphasis on the human microbiome and its relationship to human health. *PLOS Computational Biology* aims to meet this challenge and place a larger focus on this important and certain-to-become-central area in the biological sciences. Clearly, many challenges remain for computational biologists in the coming years.

*PLOS Computational Biology*, along with the computational biology community and the International Society for Computational Biology (ISCB), are poised to take on this challenge. We view this collection as the first in this direction, helping the community toward this aim.
